# Mesenchymal stem cells overexpressing IL-35: a novel immunosuppressive strategy and therapeutic target for inducing transplant tolerance

**DOI:** 10.1186/s13287-018-0988-9

**Published:** 2018-09-26

**Authors:** Hao Guo, Baozhu Li, Wei Wang, Na Zhao, Haopeng Gao

**Affiliations:** 1 Department of General Surgery, Tianjin Medical University General Hospital, No. 154 Anshan Road, Heping District, Tianjin, 300052 China; 2 Tianjin General Surgery Institute, Tianjin Medical University General Hospital, No. 154 Anshan Road, Heping District, Tianjin, 300052 China

**Keywords:** Interleukin-35, MSCs, Exosomes, Gene modification, Transplant tolerance

## Abstract

Inducing donor-specific immunological tolerance, which avoids the complications of long-term immunosuppression, is an important goal in organ transplantation. Interleukin-35 (IL-35), a cytokine identified in 2007, is mainly secreted by regulatory T cells (Tregs) and is essential for Tregs to exert their maximal immunoregulatory activity in vitro and in vivo. A growing number of studies show that IL-35 plays an important role in autoimmune diseases and infectious diseases. Recent research has shown that IL-35 could effectively alleviate allograft rejection and has the potential to be a novel therapeutic strategy for graft rejection. With increasing study of immunoregulation, cell-based therapy has become a novel approach to attenuate rejection after transplantation. Mesenchymal stem cells (MSCs), which exhibit important properties of multilineage differentiation, tissue repair, and immunoregulation, have recently emerged as attractive candidates for cell-based therapeutics, especially in transplantation. Accumulating evidence demonstrates that the therapeutic abilities of MSCs can be amplified by gene modification. Therefore, researchers have constructed IL-35 gene-modified MSCs and explored their functions and mechanisms in some disease models. In this review, we discuss the potential tolerance-inducing effects of MSCs in transplantation and briefly introduce the immunoregulatory functions of the IL-35 gene-modified MSCs.

**Electronic supplementary material:**

The online version of this article (10.1186/s13287-018-0988-9) contains supplementary material, which is available to authorized users.

## Background

Organ transplantation is one of the most effective treatments for end-stage organ failure, but the long-term survival of grafts is limited by graft rejection. The principal method of inhibiting allograft rejection is the use of immunosuppressive drugs that lack antigen specificity, such as cyclosporine, azathioprine, and sirolimus [1]. Although these immunosuppressive drugs have largely improved the prognosis of organ transplant patients, their lifelong usage leads to many adverse effects, including nephrotoxicity, opportunistic infections, diabetes, and even malignancy, which limits organ transplantation [2]. Therefore, how to reduce the complications of drugs and induce immune tolerance after transplantation is of critical significance to organ transplant patients. In recent decades, with the deepening study of immunoregulation, cell-based therapy has become a novel approach to attenuate rejection after transplantation. In particular, because of their plasticity, migratory ability, paracrine activity, immune modulatory, and regenerative properties, mesenchymal stem cells (MSCs) have recently emerged as attractive candidates for cell-based therapeutics, especially in transplantation. There is evidence that the therapeutic abilities of MSCs can be amplified by gene modification, e.g., IL-10 [3], hepatocyte growth factor [4], GATA-4 [5], and IL-35 [6]. In this review, we provide a brief overview of the potential tolerance-inducing effects of MSCs in transplantation and summarize the immunoregulatory function of the IL-35 gene-modified MSCs (IL-35-MSCs).

### IL-35: a new immunomodulator in autoimmune diseases and transplantation

IL-35, a cytokine identified in 2007, belongs to the interleukin-12 cytokine family and shares a similar structure with other members, including IL-12, IL-23, and IL-27 [7]. Each member is composed of a heterodimer of an α chain (p19, p28, or p35) and a β chain (p40 or Epstein-Barr virus-induced gene 3, EBI3), which in the case of IL-35 are p35 and EBI3, respectively. Unlike other members, IL-35 is not primarily produced by antigen-presenting cells, such as macrophages, monocytes, and dendritic cells; it is secreted primarily by CD4^+^ Foxp3^+^ regulatory T cells (Tregs) [7]. Moreover, activated B cells, activated endothelial cells, smooth muscle cells, and monocytes are also sources of IL-35 [8]. In addition to coming from different sources, the function of IL-35 is also distinct from the other members. In contrast to the pro-inflammatory effect of other cytokines (IL-12, IL-23), IL-35 is a powerful immunosuppressive cytokine that is essential for Tregs to exert their maximal immunoregulatory activity in vitro and in vivo [7]. IL-35 can inhibit the promotion of T helper (Th)1 and Th17 cell differentiation and function and can have an essential role in the balance between Th17 cells and Treg cells [9]. Interestingly, IL-35 can induce the transformation of conventional T cells (Tconv) into CD4^+^ Foxp3^−^ regulatory T cells (iTr35) that can secrete IL-35 [10]. This positive feedback cascade amplification effect greatly inhibits the effects of multiple effector cells and cytokines [11]. Recent studies have shown that IL-35-producing B cells are novel key players in the negative regulation of immunity, and regulatory B cells (Bregs) induced by IL-35 could suppress autoimmune disease by secreting IL-10 and IL-35 [12, 13]. As such, IL-35 has gradually emerged as a unique therapeutic target for autoimmune diseases and infectious diseases. In a mouse model of collagen-induced arthritis, IL-35 effectively inhibited the expression of IL-17 and attenuated the symptoms of arthritis [14] in an IL-10-dependent manner [15]. IL-35 production by B cells may be a novel therapeutic target for experimental autoimmune encephalomyelitis [13, 16]. IL-35 also plays an important role in the development of inflammatory bowel disease and significantly relieved the symptoms of colitis in mice [17]. In addition, recent studies have shown that it also has a therapeutic effect on experimental autoimmune uveitis in mice [12].

Moreover, the role of IL-35 has been gradually recognized in both autoimmune diseases and transplantation. Liu et al. [18] revealed that overexpression of IL-35 prevented the development of both immunological and clinical manifestations of acute graft-versus-host disease (aGVHD). In vivo, they found that overexpression of IL-35 suppresses CD4^+^ effector T-cell activation, leading to a reduction in alloreactive T-cell responses and aGVHD severity. IL-35 also induces the amplification of Tregs in the aGVHD target organs. Through the analysis of serum IL-35 levels from allogeneic hematopoietic stem cell transplantation (allo-HSCT) patients, the clinical relevance of IL-35 expression in aGVHD patients was determined: IL-35 was negatively related to the severity of the disease. In another study, Yin et al. [19] isolated islet cells of BALB/c mouse and purified CD4^+^ T-cell subsets of a C57BL/6 mouse to establish a model of islet transplantation in vitro by co-culture of the cells. After administration of IL-35, the number of Tregs increased significantly compared to the control group, whereas the proliferation rate of Th17 cells was significantly inhibited. Utilizing a sugar stimulation experiment, they demonstrated that IL-35 mitigates the function of murine transplanted islet cells via regulation of the Th17/Treg ratio. Our research [20] exploited a heterotopic abdominal heart transplantation model in C57BL/6 mice to investigate the role of IL-35 in allograft rejection. The results indicated that IL-35 alleviated allograft rejection, and the possible mechanism may act through enhancing the proliferation of CD4^+^ CD25^+^ Tregs and restraining the proliferation and function of effector T cells. In conclusion, the above studies show that IL-35 has the potential to be a novel therapeutic strategy for various autoimmune diseases and graft rejection.

### MSC-based therapeutics and transplant tolerance

MSCs, also known as multifunctional stromal cells or mesenchymal stromal cells, are one of the most easily accessible multipotential stem cells that can be harvested from various tissues, such as bone marrow, placenta, umbilical cord blood, adipose tissue, amniotic fluid, dental pulp, and other sources [21–23]. As a kind of multipotent stem cell, MSCs gradually emerged as a promising candidate for cell therapy in preclinical and clinical trials because of their availability and multiple biological functions, including multilineage differentiation, tissue-repair, anti-inflammatory mechanisms, immunosuppression, and neuroprotection [24]. Over the past two decades, a large amount of research has revealed that MSCs possess extensive immunoregulatory capabilities. Accumulated data provide compelling evidence that MSCs can inhibit the proliferation and function of T cells [25–27] and various professional antigen-presenting cells, including B cells, dendritic cells, and macrophages, as well as natural killer cells [28]. In addition, research shows that the proliferation and differentiation of Tregs can be induced by MSCs; the mechanisms employed by MSCs to inhibit effector T-cell proliferation overlap with the mechanisms involved in Treg induction, yet they do not interfere with Treg function, so these advantages make them a unique immunomodulator [29, 30]. The curative effects of MSCs have also been seen in various autoimmune diseases, including arthritis [31], Crohn disease [32], multiple sclerosis [33], myocardial infarction [34], and diabetes [35]. In addition, some recent studies have shown that MSCs also play an important role in transplantation.

In 2002, Bartholomew et al. [36] observed that MSCs could suppress lymphocyte proliferation in vitro and in vivo. Administration of MSCs led to prolonged skin graft survival in baboons when compared to control animals (11.3 ± 0.3 days vs 7 ± 0 days). MSCs are being gradually used in organ transplantation, and many rodent models of allogeneic heart transplantation have been used to explore the immune regulation of MSCs in vivo. In one study, Lewis rats and ACI rats were used as heart transplant donors and recipients, respectively, and MSC (bone marrow-derived MSCs from donor or recipient) injections were performed after operation [37]. MSC injections failed to demonstrate prolongation of graft survival; what is more, concurrent treatment with low-dose cyclosporine A (CsA) and MSCs accelerated allograft rejection [37]. In contrast, in a similar study, recipient Fisher344 rats were transplanted with hearts from inbred Wistar rats, and MSCs (Wistar rat BM-derived MSCs) were administered before and after transplantation. The results indicated that MSCs prolonged the survival of the grafts compared with the control group (12.4 days vs 6.4 days), and allograft tolerance may be induced by changing the Th1/Th2 balance [38]. Coincidentally, Popp et al. [39] reported that a combination of donor-derived MSCs with low-dose mycophenolate induced long-term allograft acceptance in a rat heart transplantation model, and the tolerogenic effect of MSCs may be partially mediated by the expression of indoleamine 2,3-dioxygenase (IDO). Studies have shown that MSCs also have a similar effect on a mouse heart transplant model. Relative to untreated recipients (7.5 days), rapamycin (Rapa) monotherapy (16.5 days) and donor-derived MSC monotherapy (14.0 days) prolonged the survival of the grafts; surprisingly, the combination therapy of MSCs and low-dose Rapa resulted in long-term heart graft survival (> 100 days) with normal histology [40]. The role of MSCs in kidney transplantation has been gradually revealed. Ge et al. [41] demonstrated that MSCs may mediate kidney allograft tolerance by the induction of Tregs and, to some extent, this is regulated by the IDO secreted by MSCs. Parallel to this finding, IDO-lentivirus-transfected MSCs (IDO-MSCs) possessed strong immunomodulatory capabilities in vivo and in vitro [42]. IDO-MSCs enhanced the expression and function of CD4^+^CD25^+^ Foxp3^+^ Treg cells and induced allograft tolerance in a rabbit model of orthotopic renal transplantation [42].

Although the efficacy of MSCs in a variety of diseases has become well known, the precise mechanism(s) of action of MSCs are still elusive and remain controversial. Research shows that in vivo administration of MSCs ameliorates disease in preclinical models but that these cells are rapidly cleared within 48 h [43]. Cell differentiation and direct tissue repair contribute minimally to the beneficial effects attributed to MSCs, and paracrine and immunomodulatory pathways are the predominant mechanisms of their in vivo effects [43]. MSC-derived exosomes contain multiple mRNAs, microRNAs (miRNAs), and proteins that could alter the ability of target cells to exert their function, such as repairing tissue damage, suppressing inflammatory responses, and modulating the immune system [24]. Timmers et al. [44] confirmed that conditioned medium of human MSCs significantly reduced infarct size in both pig and mouse models of myocardial ischemia/reperfusion (MI/R) injury, but the active component and the mechanism of action have not been determined. In another study, utilizing a mouse model of MI/R injury, researchers initially investigated the function of MSC-derived exosomes. The results showed that MSC-derived exosomes reduced infarct size, and the MSCs mediated their cardioprotective paracrine effect by secreting exosomes [45]; then, MSC-derived exosomes were applied to several disease models. For example, the nephrotoxicity of cisplatin can be effectively relieved via the activation of autophagy induced by human umbilical cord MSC-derived exosomes [46]. Intramuscular injection of MSC-derived exosomes markedly promoted angiogenesis in mouse ischemic limbs, effectively attenuating ischemic injury [47]. Gong et al. [48] suggested that angiogenesis may be mediated by MSC-derived exosomes by transferring pro-angiogenic miRNAs to endothelial cells. There is also evidence that MSC-derived exosomes have potential immunomodulatory abilities such as MSCs. Yang et al. [49] indicated that MSC-derived exosomes could relieve symptoms in a rat colitis model by reducing the level of pro-inflammatory cytokines, inhibiting NF-ĸBp65 signal transduction pathways, modulating the antioxidant/oxidant balance, and affecting the occurrence of apoptosis. They also have the potential to attenuate an activated immune system through the induction of IL-10 and Tregs, and MSC-derived exosome administration significantly improved skin allograft survival [50]. There was also a clinical case in which MSC-derived exosomes had therapeutic potential in GVHD [51]. Compared with the cytokine responses of the patient’s peripheral blood mononuclear cells (PBMCs) before MSC-derived exosome therapy, the numbers of IL-1β, TNF-α, and IFN-γ-producing PBMCs were reduced by more than 50% after exosome application. The symptoms of cutaneous and mucosal GVHD were remarkably ameliorated within 2 weeks, and the effects lasted for 4 months. Owing to the clinical response, the dosage of the steroids could be reduced from 125 mg/d before to 30 mg/d after the MSC-derived exosome therapy.

In addition, the cumulative evidence indicates that preconditioning or genetic manipulation of the parent cells can markedly improve the therapeutic effect of exosomes. For example, compared with the control group, exosomes from MSCs transduced with lentiviral CXCR4 showed a better efficiency for reducing left ventricular remodeling and promoting restoration of heart function after myocardial infarction via the Akt signaling pathway [52]. In another study, researchers found that exosomes derived from MSCs overexpressing GATA-4 could exert more cardioprotective effects by delivering miRNAs to regulate the target proteins in recipient cells [53]. MiR-122-transfected MSCs can effectively package miR-122 into secreted exosomes, and intra-tumor injection of exosomes derived from miR-122-modified MSCs significantly increased the antitumor efficacy of sorafenib on hepatocarcinoma in vivo [54].

Although MSCs have recently emerged as promising therapeutics for the improvement of GVHD, autoimmune disease, the severity of cardiovascular disease, and anti-transplant rejection, the main candidate for clinical application is still bone marrow-derived MSCs (BM-MSCs) [55]. However, BM-MSCs usually cannot guarantee long-term immunomodulatory effects in vitro and in vivo [56]. In addition, the clinical application of MSCs often requires a large number of cells, while the number of MSCs obtained from a single donor is limited. As an important aspect of cell therapy, the discovery of human-induced pluripotent stem cells (hiPSCs) greatly promoted the development of regenerative medicine. On the one hand, hiPSCs are customized and infinitely expandable in vitro and thus offer an unlimited source for MSC generation; on the other hand, patient-specific MSCs derived from hiPSCs (iMSCs) can be used for autologous transplantation without the need for immunosuppression [57]. Moreover, because of their better cellular vitality, such as survival, proliferation, and differentiation potentials [58], autologous iMSCs probably serve as an inexhaustible source of MSCs that could be used to meet growing clinical requirements [59]. For example, Himeno et al. [60] revealed that iMSCs ameliorate diabetic polyneuropathy in mice, and they might exert therapeutic effects on diabetic polyneuropathy by secreting angiogenic/neurotrophic factors and differentiating into Schwann cell-like cells. Lian et al. [57] indicated that iMSCs can be clonally generated, beginning at the single-cell level, from hiPSCs. Limb ischemia in mice can be attenuated by iMSCs. The mechanism by which iMSCs outperform MSCs may be due to their superior survival and engraftment after transplantation and their ability to induce vascular and muscle regeneration via direct de novo differentiation and paracrine mechanisms [57]. In another study, Giuliani et al. [56] suggested that iMSCs can be used as an effective treatment to prevent allograft rejection, and their capacity to impair NK cell cytotoxicity constitutes a potential mechanism of action. In addition, although there are certain technical and regulatory hurdles, some scholars believe that the combination of a proven gene therapy with iMSCs might hold great therapeutic potential [59], and the discovery of iMSCs could provide a broader clinical perspective for MSC-based therapeutics.

In brief, as a highly promising candidate for stem cell-based therapy, MSCs, especially genetically modified MSCs, may have powerful immunosuppressive capabilities. Exosomes derived from genetically pre-treated MSCs may have great potential to become ideal vehicles for cell-free therapy. In recent years, researchers have constructed MSCs with modified IL-35 gene expression (IL-35-MSCs), preliminarily exploring their function and mechanism of action in some disease models [6, 61–63]. In addition, the exosomes secreted by IL-35-MSCs have become an important research topic.

### IL-35-MSCs: a novel gene therapy strategy

As a promising candidate for cell-based immune tolerance therapy, IL-35-MSCs have been researched due to their advantages, such as ease of availability, the ability to express IL-35 steadily and continuously in vivo and in vitro, and stronger immunosuppressive effects than MSCs. Zhao et al. [6] initially reported the immunosuppressive function of IL-35-MSCs in vitro. First, adipose tissue-derived MSCs were isolated from male C57BL/6 J mice (4–5 weeks old) and transfected with a lentivirus vector for the overexpression of the therapeutic murine IL-35 gene, and IL-35-MSCs could express and secrete IL-35 successfully in vitro. Next, they studied the functions of IL-35-MSCs in vitro via co-culture experiments, and the results indicated that IL-35-MSCs significantly inhibited the proliferation of CD4^+^ T cells. Increased IL-10 and decreased IL-17 was produced by CD4^+^ T cells in the presence of IL-35-MSCs. In addition, relative to the control group (cells co-cultured with MSCs or alone), IL-35-MSCs increased the proportion of CD4^+^ Foxp3^+^ Tregs. It has become a consensus that the Tregs induced by IL-35 are Foxp3^−^, and the mechanism of IL-35-MSCs outperforming MSCs in the induction of Foxp3^+^ Tregs is still unknown. In in vivo experiments, our results [61] revealed that IL-35-MSCs could enhance the proliferation of CD4^+^ CD25^+^ Treg cells and suppress the function of effector T cells, such as Th1, Th2, and Th17 cells, in vivo. Using spleen mononuclear cells collected from BALB/c mice and IL-35-MSC-treated C57BL/6 mice as stimulator cells and responder cells, respectively, a one-way mixed lymphocyte reaction was induced to evaluate the effects of IL-35-MSCs on murine immune function. The results showed that IL-35-MSCs stimulated proliferation of CD4^+^ CD25^+^ Treg cells and inhibited the proliferation of CD4^+^ T cells, identified the effects of IL-35-MSCs on allograft rejection, and suggested that IL-35-MSCs could be a potential target for cell therapy in solid organ transplantation. Moreover, IL-35-MSCs also played an important role in the prevention of autoimmune diseases. Another study [62] investigated the protective effects of IL-35-MSCs in concanavalin A (Con A)-induced autoimmune hepatitis. First, C57BL/6 J mice were injected intravenously with Con A to induce hepatitis, and then they were divided into three groups and intravenously injected with IL-35-MSCs, MSCs, or phosphate-buffered saline (PBS). After the observation period (72 h), all the mice transplanted with IL-35-MSCs survived, while the PBS pre-treated mice died within 24 h, and the survival rate of MSC-transplanted mice was 40%. Further research indicated that IL-35-MSCs could specifically migrate to the injured liver tissues and significantly decrease hepatocyte necrosis and apoptosis by reducing FASL expression by mononuclear cells. Relative to MSCs, the mechanism of IL-35-MSCs having a better treatment effect is probably because IL-35-MSCs decreased the level of IFN-γ secreted by liver mononuclear cells, which may be achieved by activating the JAK1-STAT1/STAT4 signaling pathway. Nevertheless, the specific mechanism of IL-35-MSCs exerting more therapeutic effects than MSCs is still uncertain. Our most recent study [63] demonstrated that IL-35-MSCs could ameliorate ulcerative colitis (UC) by down-regulating the expression of pro-inflammatory cytokines. In this study, dextran sulfate sodium was used to induce colitis in mice, and the mice were treated with IL-35-MSCs, MSCs, or saline. The results showed that mice in the two treated groups recovered their body weight more rapidly than mice treated with saline in the later stage of colitis. The colons of IL-35-MSC-treated mice were markedly longer than those in the other two groups, and inflammation was reduced significantly. In addition, IL-35-MSCs increased the percentage of Foxp3^+^ Tregs and decreased the level of pro-inflammatory cytokines (TNF-α, IFN-γ and IL-17) produced by lamina propria lymphocytes significantly. In conclusion, this study revealed that IL-35-MSCs may represent an attractive therapeutic strategy for the treatment of UC.

## Conclusions

In organ transplantation, allograft rejection is a major obstacle to the long-term survival of transplanted organs, and establishing donor-specific immunological tolerance, which avoids the complications of long-term immunosuppression (infections, malignancies, cardiovascular disease, renal failure, etc.), has long been an important goal. Although significant progress has been achieved in the development of approaches to the treatment of anti-transplant rejection, the mechanism of inducing transplant tolerance remains obscure. However, in recent decades, with the increased study of immunoregulation, cell-based therapy has become a novel approach to attenuate rejection after transplantation.

IL-35, a cytokine identified in 2007, belongs to the IL-12 cytokine family and is composed of the Epstein-Barr virus-induced gene 3 and p35 subunits [7]. IL-35 is secreted primarily by CD4^+^ Foxp3^+^ Tregs and is essential for Tregs to exert their maximal immunoregulatory activity in vitro and in vivo [7]. Interestingly, IL-35 can induce Tconv into iTr35, which can secrete IL-35 [10]. This positive feedback cascade amplification effect greatly inhibits the effects of multiple effector cells and cytokines [11]. Previous studies on IL-35 mainly focused on autoimmune diseases [12–17], but recent research has shown that IL-35 could effectively alleviate allograft rejection and has the potential to be a novel therapeutic strategy for graft rejection [18–20].

In recent years, MSCs have been promising candidates for cell-based immunotherapy in preclinical and clinical trials. Research indicates that MSCs are effective in many diseases, such as arthritis, Crohn’s disease, multiple sclerosis, myocardial infarction, diabetes, and systemic lupus erythematosus [31–35]. In addition, studies have shown that MSCs can also efficaciously alleviate graft rejection in a variety of animal models and play an important role in transplantation [36–42], but the specific mechanism is ambiguous. Accumulating evidence supports the notion that MSCs act in a paracrine manner, and the exosomes derived from MSCs have functions similar to those of MSCs as one of the mechanisms of its paracrine function [43]. MSC-derived exosomes contain multiple mRNAs, miRNAs, and proteins that could alter the activity of target cells to exert their function, such as repairing tissue damage, suppressing inflammatory responses, and modulating the immune system [24]. What is more exciting is that the function of MSCs and their exosomes can be improved by preconditioning or genetic modification [3–6, 42, 52–54]. Based on these findings, the researchers constructed IL-35-MSCs and explored their function and mechanism of action in some disease models [6, 61–63]. Research shows that IL-35-MSCs can continuously and stably secrete IL-35 in vivo and in vitro, exerting a stronger immunosuppressive effect than MSCs [6]. They can effectively relieve the symptoms of Con A-induced fulminant hepatitis via effectively inhibiting proliferation and function of CD4^+^ T cells and up-regulating CD4^+^ Foxp3^+^ Tregs [62]. They also have the potential to be a promising and attractive cell therapy approach for autoimmune diseases [63] and allograft rejection [61]. Researchers have reached a consensus that Tregs induced by IL-35 are iTr35 and do not express Foxp3 [10], and the mechanism by which IL-35-MSCs outperform MSCs in inducing Foxp3^+^ Treg cells is still unknown. However, in view of the accumulating evidence that the characteristics of MSC-derived exosomes can be changed by gene modification [52–54], it is reasonable to hypothesize that IL-35 gene modification may change the content (such as cytokines and growth factors, signaling lipids, mRNAs, and miRNAs) of MSC-derived exosomes, which further induces the differentiation of Foxp3^+^ Treg cells. An additional file shows this in more detail (see Additional file 1). Compared with cell therapy, exosomes are more stable, have lower immunogenicity and can reach higher treatment doses. We hypothesize that with further research on their functions and mechanisms, IL-35-MSCs and their exosomes may become a feasible and promising therapeutic target in various diseases and transplantation tolerance. In conclusion, compared with MSCs, IL-35-MSCs may exert stronger immunosuppression effects, which has broad research potential and value for various autoimmune diseases and organ transplantation.

## Additional file

Additional file 1: The hypothetical mechanism of IL-35-MSCs for regulation of the immune response. Description: IL-35 secreted by IL-35-MSCs could induce Tconv to differentiate into iTr35, which can secrete IL-35. This positive feedback cascade amplification effect ensures the continuous and stable expression of IL-35 in vivo or vitro and inhibits the effects of multiple effector cells and cytokines. IL-35 gene modification may change the content (such as cytokines and growth factors, mRNAs, and miRNAs) of MSC-derived exosomes, which further induces the differentiation of Foxp3^+^ Treg cells, thus exerting a stronger immunosuppressive effect than MSCs in vivo or vitro. (JPG 44 kb)

## Abbreviations

aGVHD: Acute graft-versus-host disease
allo-HSCT: Allogeneic hematopoietic stem cell transplantation
BM-MSC: Bone marrow-derived MSC
Breg: Regulatory B cell
Con A: Concanavalin A
CsA: Cyclosporine A
EBI3: Epstein-Barr virus-induced gene 3
hiPSC: Human-induced pluripotent stem cell
IDO: Indoleamine 2,3-dioxygenase
IDO-MSC: IDO-lentivirus-transfected mesenchymal stem cell
IL-35: Interleukin-35
IL-35-MSC: IL-35 gene-modified mesenchymal stem cell
iMSC: MSC derived from human-induced pluripotent stem cells
iTr35: IL-35-producing CD4^+^ Foxp3^−^ -induced regulatory T-cell population
MI/R injury: Myocardial ischemia/reperfusion injury
miRNA: MicroRNA
MSC: Mesenchymal stem cell
PBMC: Peripheral blood mononuclear cell
PBS: Phosphate-buffered saline
Rapa: Rapamycin
Tconv: Conventional T cell
Th cell: T helper cell
Treg: Regulatory T cell
UC: Ulcerative colitis
