# Supplementary figures and images for: Mesenchymal stem cells overexpressing IL-35: a novel immunosuppressive strategy and therapeutic target for inducing transplant tolerance
Source: Stem Cell Res Ther. 2018 Sep 26;9:254. doi: 10.1186/s13287-018-0988-9 (PMC6158805; doi:10.1186/s13287-018-0988-9)

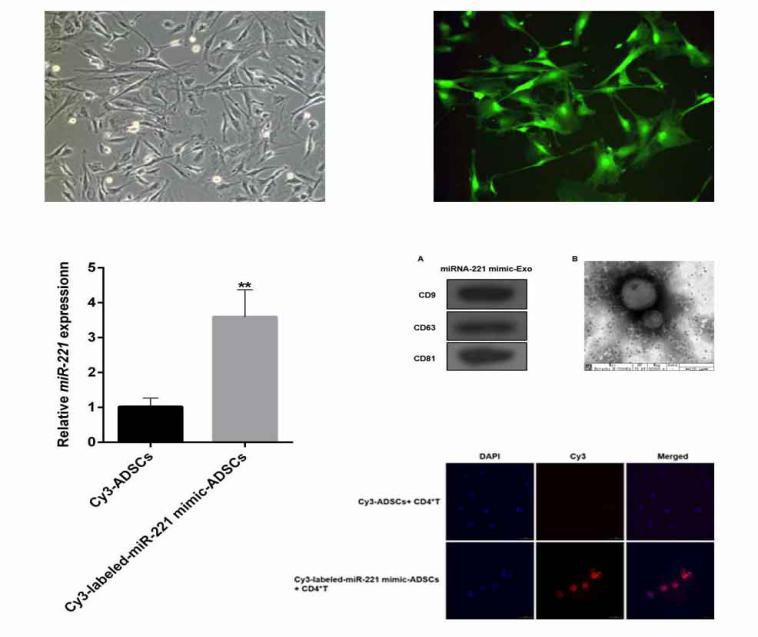

Supplement: Supplementary file 1 — The hypothetical mechanism of IL-35-MSCs for regulation of the immune response. Description: IL-35 secreted by IL-35-MSCs could induce Tconv to differentiate into iTr35, which can secrete IL-35. This positive feedback cascade amplification effect ensures the continuous and stable expression of IL-35 in vivo or vitro and inhibits the effects of multiple effector cells and cytokines. IL-35 gene modification may change the content (such as cytokines and growth factors, mRNAs, and miRNAs) of MSC-derived exosomes, which further induces the differentiation of Foxp3+ Treg cells, thus exerting a stronger immunosuppressive effect than MSCs in vivo or vitro. (JPG 44 kb) [file 13287_2018_988_MOESM1_ESM.jpg]
